# Outcomes in acute ischemic stroke patients undergoing endovascular thrombectomy: Cervical internal carotid artery pseudo-occlusion vs. true occlusion

**DOI:** 10.3389/fneur.2022.1106358

**Published:** 2023-01-09

**Authors:** Heng Ni, Tao Yang, Zhenyu Jia, Haibin Shi, Sheng Liu, Linbo Zhao

**Affiliations:** ^1^Department of Interventional Radiology, The First Affiliated Hospital of Nanjing Medical University, Nanjing, China; ^2^Department of Neurology, Changzhou Second People's Hospital, Changzhou, China

**Keywords:** pseudo-occlusion, endovascular thrombectomy, acute ischemic stroke, internal carotid artery, outcome

## Abstract

**Background and purpose:**

Pseudo-occlusion of the cervical internal carotid artery (cICA) refers to an absence of intraluminal contrast on computed tomography angiography (CTA), while the artery is patent on digital subtraction angiography during endovascular thrombectomy. We aimed to compare the outcomes between the cICA pseudo-occlusion and true occlusion after thrombectomy.

**Methods:**

We retrospectively analyzed patients with apparent cICA occlusion on CTA who underwent thrombectomy between January 2016 and August 2021, and divided them into the pseudo-occlusion and true occlusion groups based on angiographic exploration. Recanalization failure was defined as a modified Thrombolysis in Cerebral Infarction score of 0–2a. Poor outcome was defined as a 90-day modified Rankin Scale score of 3–6. Propensity score matching was performed to compare the outcomes. Sensitivity analysis using multivariate-adjusted regression in the original cohort was conducted to test the robustness of the findings.

**Results:**

Of the 146 patients included, 79 patients (54.1%) had cICA pseudo-occlusion and 67 patients (45.9%) had true occlusion. Following 1:1 propensity score-matched analysis, patients with pseudo-occlusion had an increased likelihood of recanalization failure (OR, 3.18; 95% CI, 1.06–9.59; *P* = 0.040) and poor outcome (OR, 2.80; 95% CI, 1.07–7.30; *P* = 0.035) compared with patients with true occlusion. Sensitivity analysis showed that cICA pseudo-occlusion remained independently associated with recanalization failure (OR, 2.55; 95% CI, 1.07–6.09; *P* = 0.036) and poor outcome (OR, 2.48; 95% CI, 1.08–5.67; *P* = 0.032).

**Conclusions:**

Patients with cICA pseudo-occlusion on CTA treated with thrombectomy had an increased risk of reperfusion failure and poor outcome compared with true occlusion patients.

## Introduction

Endovascular thrombectomy has been accepted as a standard approach in patients with acute ischemic stroke caused by intracranial large vessel occlusions (1). Computed tomography angiography (CTA) is an established imaging modality in determining the location of vascular occlusions, but the misinterpretation of initial CTA images is common in clinical practice (2). Several studies have reported the existence of cervical internal carotid artery (cICA) pseudo-occlusion (PO), an absence of intraluminal contrast material on CTA but the carotid artery is patent on digital subtraction angiography (DSA) (3–6). This phenomenon is a flow-related artifact that mimics cICA true occlusion on single arterial-phase CTA, which is likely the result of the sluggish or absent contrast penetration caused by distal intracranial artery occlusion.

The mechanism of occlusion and the subsequent thrombectomy planning setting for PO and true occlusion of the cICA are completely different, which may affect reperfusion and prognosis (7, 8). Previous publications have demonstrated the clinical impact of PO phenomenon in the populations with distal ICA occlusion or isolated middle cerebral artery (MCA) occlusion (9–11). However, the control groups in the reports above were patients with non-PO, and no analysis was conducted to compare the outcomes after thrombectomy between patients with PO and true occlusion. Therefore, this study aimed to compare the characteristics, reperfusion rates, and clinical outcomes between the cICA PO and true occlusion groups.

## Materials and methods

### Patient selection

**Figure 1:**
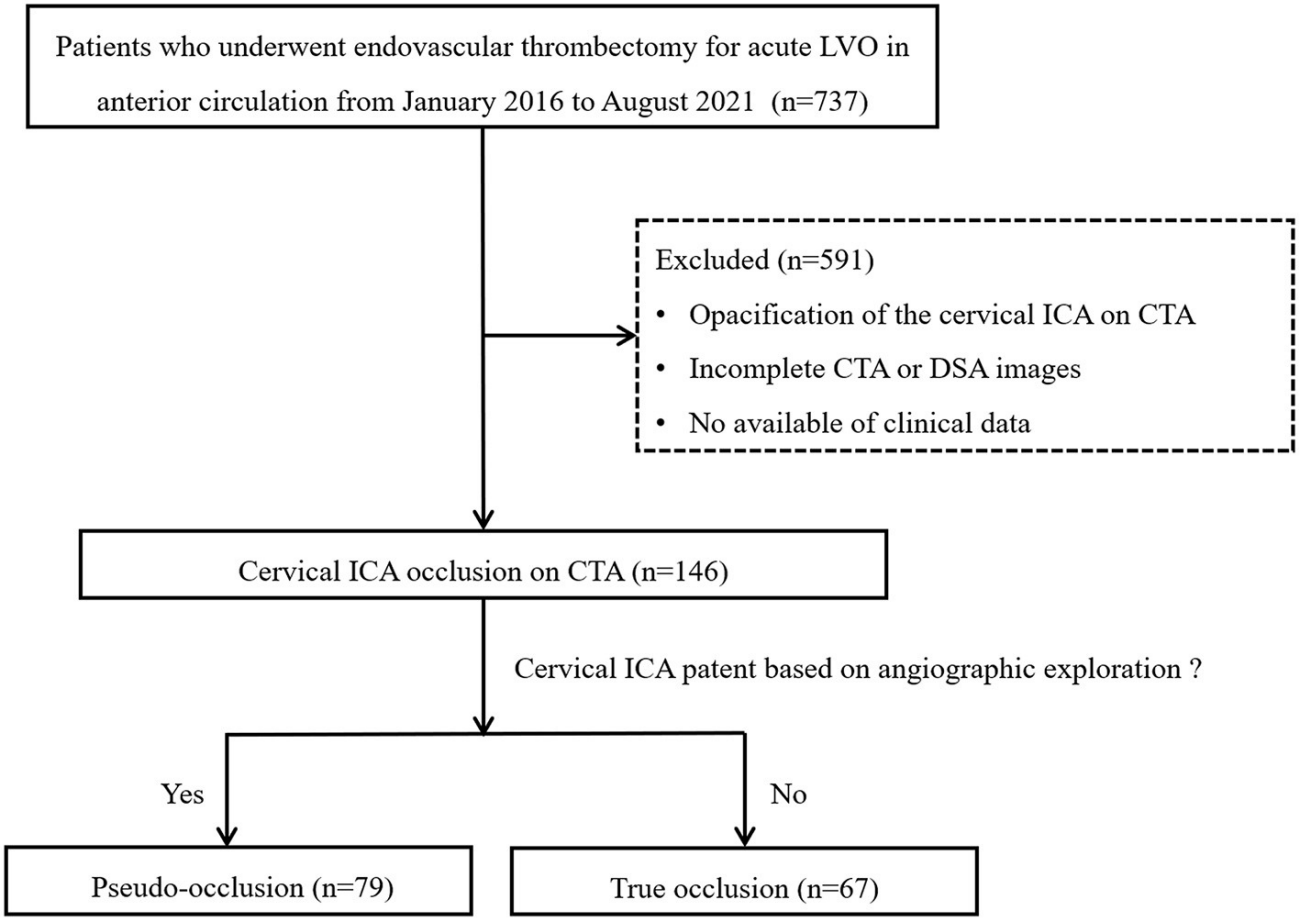
Flowchart of patient selection. LVO, large vessel occlusion; ICA, internal carotid artery; CTA, computed tomography angiography; DSA, digital subtraction angiography.

Between January 2016 and August 2021, we retrospectively reviewed the clinical data of 737 consecutive patients who underwent thrombectomy for acute large vessel occlusion in anterior circulation based on the stroke database. The inclusion criteria were as follows: (1) age of ≥18 years; (2) an initial National Institutes of Health Stroke Scale (NIHSS) score of ≥6; (3) a modified Rankin scale (mRS) score of ≤ 2 before acute stroke; (4) Alberta Stroke Program Early Computed Tomography Scores (ASPECT) of ≥ 6 within 6 h of symptom onset; (5) fulfillment of DAWN or DEFUSE-3 criteria in computerized tomography perfusion (CTP) imaging within 6 to 24 h of last known normal; and (6) non-visualization of the cICA on single arterial-phase CTA or artery peak phase of 3-phase-CTA reconstructed from CTP. The following patients were excluded: (1) incomplete CTA or DSA images; and (2) loss of clinical data or follow-up results. The detailed flow diagram of the patient selection is shown in Figure 1. This study was approved by the local institutional review board, and because of the retrospective study design, the requirement for informed consent from patients was waived.

### Image acquisition and analysis

Computed tomography (CT) scans including non-contrast CT, CTA, and CTP by a 128-slice multidetector CT scanner (optima CT 660, GE Medical Systems, Chicago, USA) were initially obtained for each patient upon admission to the emergency department. Before December 2019 in our institution, patients with onset time < 5 h underwent non-contrast CT and CTA. Non-contrast CT was acquired using the axial technique with 120 kVp, 100–350 auto-mAs, and 5-mm section thickness reconstructions. CTA was performed from the aortic arch to the vertex; contrast material was administered by a power injector at 4 mL/s into an antecubital vein. Source images (CTA-SI) were reconstructed to 0.625-mm thickness at 0.625-mm intervals. The first phase of simulated multiphase CTA, which was also used as simulated single-phase CTA, was reconstructed from the peak arterial phase in the normal distal ICA, based on the arterial input function curve. CTP was performed in patients with onset time ≥ 5 h or with unknown onset time and was conducted using a periodic spiral approach (4-dimensional adaptive spiral mode, 100 kVp, 200 mAs, rotation time 0.4 s, maximum pitch 0.984). Post-processing of CTP data was performed by using a commercially available software named F-STROKE (Neuroblem, Shanghai, China). After semi-automated motion correction, parametric colored CTP maps were automatically obtained. The single-phase CTA was reconstructed from CTP data, with the peak arterial phase in the normal distal ICA based on the arterial input function curve. The stroke imaging protocol after December 2019 was changed as follows: all patients suspected of acute ischemic stroke underwent non-contrast CT and CTP scans. Perfusion images were post-processed with commercial software RAPID, and the single-phase CTA was reconstructed from CTP data, with the peak arterial phase in the normal distal ICA based on the arterial input function curve. A slice thickness of 0.625 mm every 1 mm was used. Single-phase CTA analysis was performed by using 24-mm axial sliding maximum intensity projection, 0.625-mm axial source images and orthogonal multiplanar reformat as needed.

**Figure 2:**
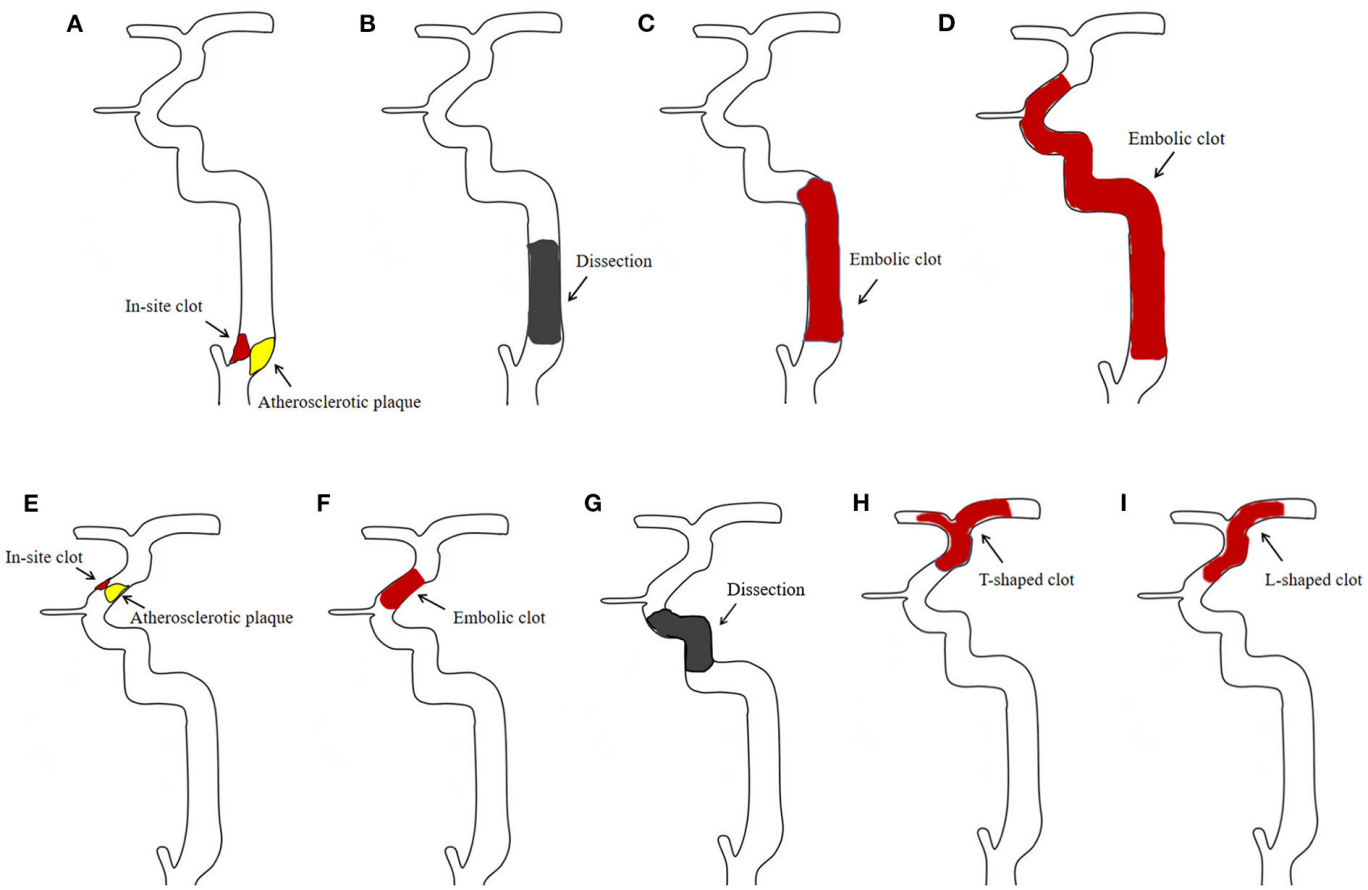
Schematic diagram of internal carotid artery (ICA) occlusions. True occlusion of the cervical ICA is caused by atherosclerosis **(A)**, dissection **(B)**, and massive thrombosis **(C, D)**. Pseudo-occlusion of the cervical ICA is caused by distal intracranial occlusion **(E–I)** with the above-mentioned etiologies.

Two interventional neuroradiologists blinded to the clinical information reviewed the CTA images to detect the occlusion of the cICA. Another neuroradiologist helped to reach a consensus agreement if there was any discrepancy between the two readers. Meanwhile, DSA images of these cases were re-examined to determine whether the proximal cICA was actually patent. Subsequently, these patients were divided into the PO and true occlusion groups. True occlusion of the cICA was defined as an obstruction that hindered the passage of contrast agent and guidewire on DSA, including ICA bulb atherosclerotic occlusion (including high-grade stenosis), cervical dissection, and cervical occlusion with massive thrombosis (Figures 2A–D). Conversely, PO was considered as a gradual contrast decay (a flame-shaped leading contrast edge) in the ICA above the level of the carotid bulb, in the absence of plaque around the bulb, and the vessel was normal based on angiographic exploration but intracranial occlusion was apparent, such as T or L-shape occlusions of terminal ICA, and occlusion of paraclinoid segment of ICA caused by plaque, dissection or massive thrombosis (Figures 2E–I).

### Thrombectomy procedure

The procedure was conducted under local anesthesia. An 8F introducer was inserted retrogradely into the femoral artery, allowing the guiding catheter to advance into the ipsilateral contralateral carotid artery. An initial angiographic run was done to identify whether the cICA was indeed occluded and to ascertain the etiology of the occlusion. If the true occlusion of the cICA was confirmed, different thrombectomy protocols were established for patient with isolated ICA occlusion and tandem ICA occlusion. For patients with isolated ICA occlusion, a 6F intermediate catheter (Navien 6F, Medtronic) was navigated until direct aspiration resistance in the proximal ICA was encountered, and the operators evaluated this lesion again. In cases of obstruction related to atherosclerosis disease, a 0.021-inch microcatheter (Headway; MicroVention, Tustin, California) was used to cross the occluded segment over a 0.014-inch microwire (PT2; 300 cm), and contrast injection with the microcatheter was performed to confirm the true lumen and patency of the downstream arteries. An embolic protection device (EPD) (Spider, Medtronic) was introduced into the distal segment of the ICA over 300 cm of microwire PT2 if the ICA distal to the occluded segment was patent, and a carotid stent (Wallstent, Boston/Precise, Cordis) was deployed immediately over the EPD wire with or without balloon angioplasty. In the event of an obstruction related to cICA dissection, a 0.021-inch microcatheter was navigated through the true lumen of the dissection over a microwire. The intermediate 6F guiding catheter was then advanced over the microcatheter into the distal ICA with a 50-cc syringe for manual aspiration. The carotid stent (Wallstent, Boston/Precise, Cordis) system was advanced to the dissection site through the intermediate catheter and deployed after withdrawing the intermediate catheter. The Leo Plus stent was used for the long segment of cICA dissection. In patients with tandem ICA occlusion, intracranial thrombectomy were first performed using an intermedia catheter through the occluded lesion prior to cICA reconstruction. The tirofiban was administered intravenously with a bolus dose of 12 μg/kg and a maintenance dose of 0.1 μg/kg/min immediately after stent deployment.

In cases of cICA PO, an aspiration catheter (React, Medtronic) was advanced into the distal ICA with synchronous manual aspiration. If aspiration failed to recanalize the occluded ICA or the thrombus moved to M1 or proximal M2, mechanical thrombectomy with a Solitaire stent (Solitaire, FR/AB, ev3, Irvine, California, USA) was performed. If residual severe intracranial ICA stenosis was identified after thrombectomy, balloon angioplasty was performed and tirofiban was administered intravenously at the above-mentioned dosage. Following recanalization, we ran further angiographic runs immediately and again at 20-min intervals. If the angiography revealed re-occlusion, stent placement was considered for atherosclerotic intracranial ICA occlusion. Reperfusion failure was defined as a modified thrombolysis in cerebral infarction (mTICI) score of 0 to 2a.

For patients who underwent stent insertion or intracranial balloon angioplasty, aspirin (100 mg) and clopidogrel (300 mg) were given orally or *via* a nasogastric tube 20 h after the procedure, with 4 h of overlap with intravenous administration of tirofiban when patients were excluded from significant intraparenchymal hemorrhage through the CT scan. For at least 3 months, the patient was given dual antiplatelet treatment. After that, a single antiplatelet therapy was continued for a year.

### Data collection and assessment

We reviewed the clinical data, including the demographic features, medical history, procedural details, and clinical outcome. The NIHSS score at admission was used to assess stroke severity. Stroke subtypes were classified according to the Trial of Org 10172 in Acute Stroke Treatment classification (12). The ASPECTS scores were obtained from baseline non-contrast CT. CT scans were performed on all patients immediately after the procedure and 24 h after. The follow-up magnetic resonance imaging, including magnetic resonance angiography and perfusion sequences, was performed ~1 week after the procedure if the patient cooperated. In case of any deterioration in the patient's neurological status, a non-contrast CT scan was performed immediately to exclude intracranial hemorrhage. Symptomatic intracranial hemorrhage (sICH) is defined as any hemorrhage leading to a deterioration in the patient's neurologic status (e.g., drowsiness, worsening of hemiparesis) with an increase of the NIHSS score of at least 4 or more points from baseline, or leading to death. Clinical outcomes were assessed using the mRS score, which was obtained from the clinic or through telephonic interviews; an mRS score of 3–6 at 90 days was considered a poor outcome.

### Statistical analysis

Continuous variables were described as means (standard deviation, SD) or medians (interquartile range, IQR) and categorical variables were presented as frequencies (%). The Shapiro-Wilk test was used to assess the normality of the distributions. A Student's *t* test or a Mann-Whitney U test was performed to analyze continuous data. The Fisher exact or χ*2* test was used to analyze categorical data. In addition, propensity score matching (PSM) analysis was used to balance the baseline characteristics between the PO and true occlusion groups. The variables including age, sex, atrial fibrillation, and baseline NIHSS score, were selected to generate the propensity score using a logistic regression model. After propensity score generation, patients in the two groups underwent 1:1 PSM with the nearest neighbor matching and a caliper width of 0.02. Each propensity score-derived matched pair was assigned a unique pair ID. The risk-adjusted estimates for reperfusion and clinical outcome were calculated after matching.

To test the robustness of the results, sensitivity analyses using multivariate-adjusted models were undertaken in the original cohort. Significant clinical factors (*p* < 0.1) identified using univariate-unadjusted models were included in the multivariate logistic analysis to determine odds ratios (ORs) and confidence intervals (CIs). The statistical significance level was set at 0.05. Statistical analyses were carried out with SPSS Statistics 24 (IBM- Armonk, New York, USA).

## Results

### Baseline characteristics and outcomes

A total of 146 participants who met the inclusion criteria were enrolled in the present analyses. Within this patient cohort, 79 patients had cICA PO (54.1%) and 67 patients had true occlusion (45.9%). Tandem occlusion was observed in 64 of 79 (81.0%) of patients with true occlusion. In addition, T or L-shape occlusions of terminal ICA were present in 46 of 67 (68.7%) patients with PO. The comparison between the PO and true occlusion groups is presented in Table 1. Patients with PO were older (mean age of 72 years vs. 65, *P* = 0.001), more likely to be female (53.2 vs. 25.4%, *P* = 0.001), had a higher incidence of atrial fibrillation (55.7 vs. 23.9%; *P* < 0.001), and had a lower incidence of large artery atherosclerotic stroke (24.1 vs. 58.2%; *P* < 0.001) compared with patients with true occlusion. In addition, those in the PO group also had a higher median admission NIHSS score (19 vs. 15; *P* = 0.013). There was no significant difference between the two groups in terms of baseline ASPECTS score and procedure details.

**Table 1:** Comparison between the cervical ICA PO and TO groups using propensity score matching.

	**Before matching**			**After matching (1:1)**		

**Variable**	**Cervical ICA PO** **(*****n*** = **79)**	**Cervical ICA TO** **(*****n*** = **67)**	***P*** **-value**	**Cervical ICA PO** **(*****n*** = **36)**	**Cervical ICA TO** **(*****n*** = **36)**	***P*** **-value**
**Baseline characteristics**						
Age (years), mean ± SD	72 (11.6)	65 (12.4)	0.001	68 (11.6)	66 (12.5)	0.450
Female sex, *n* (%)	42 (53.2)	17 (25.4)	0.001	11 (30.6)	15 (41.7)	0.326
Hypertension, *n* (%)	54 (68.4)	38 (56.7)	0.147	20 (55.6)	22 (61.1)	0.633
Diabetes mellitus, *n* (%)	18 (22.8)	18 (26.9)	0.569	8 (22.2)	12 (33.3)	0.293
Atrial fibrillation, *n* (%)	42 (53.2)	16 (23.9)	< 0.001	14 (38.9)	15 (41.7)	0.810
Coronary heart disease, *n* (%)	16 (20.3)	11 (16.4)	0.552	9 (25.0)	6 (16.7)	0.384
Hyperlipidemia, *n* (%)	5 (6.3)	9 (13.4)	0.146	3 (8.3)	6 (16.7)	0.476
History of stroke, *n* (%)	12 (15.2)	12 (17.9)	0.659	3 (8.3)	3 (8.3)	1.000
Smoking, *n* (%)	16 (20.3)	20 (20.3)	0.180	13 (36.1)	8 (22.2)	0.195
Stroke etiology, *n* (%)			< 0.001			0.577
LAA	19 (24.1)	39 (58.2)		14 (38.9)	14 (38.9)	
Cardio-embolism	57 (72.1)	22 (32.8)		21 (58.3)	19 (52.8)	
Dissection	3 (3.8)	6 (9.0)		1 (2.8)	3 (8.3)	
Intravenous rtPA, *n* (%)	27 (34.2)	22 (32.8)	0.864	13 (36.1)	10 (27.8)	0.448
Baseline ASPECTS, median (IQR)	7 (6–8)	7 (6–9)	0.055	7 (6–8)	7 (6–8)	0.743
Baseline NIHSS, median (IQR)	19 (13–24)	15 (11–21)	0.013	17 (12–22)	16 (13–23)	0.608
Early time windows, *n* (%)	63 (79.7)	57 (85.1)	0.402	30 (83.3)	32 (88.9)	0.733
OTP time (min), median (IQR)	248 (183–341)	251 (190–315)	0.883	257 (189–334)	234 (171–290)	0.883
Procedure time (min), median (IQR)	80 (49–101)	85 (67–124)	0.105	69 (50–102)	80 (68–124)	0.105
**Outcome characteristics**						
Successful recanalization, *n* (%)	55 (69.6)	57 (85.1)	0.028	22 (61.1)	30 (83.3)	0.035
Hemorrhagic transformation, *n* (%)	39 (49.4)	31 (46.3)	0.709	21 (58.3)	21 (58.3)	1.000
sICH, *n* (%)	15 (19.0)	6 (9.0)	0.085	8 (22.2)	5 (13.9)	0.358
3-month mRS score of 3–6, n (%)	51 (64.6)	28 (41.8)	0.006	24 (66.7)	15 (41.7)	0.034
Mortality at 90 days, *n* (%)	25 (31.6)	10 (14.9)	0.018	12 (33.3)	8 (22.2)	0.293

ICA, internal carotid artery; PO, pseudo-occlusion; TO, true occlusion; LAA, large arterial atherosclerosis; rtPA, recombinant tissue plasminogen activator; ASPECTS, Alberta Stroke Program Early CT Score; NIHSS, National Institutes of Health Stroke Scale; OTP, onset to puncture; sICH, symptomatic intracranial hemorrhage; mRS, modified Rankin scale.

Patients in the PO group had a higher likelihood of recanalization failure (30.4 vs. 14.9%; *P* = 0.028) and unfavorable outcome (64.6 vs. 41.8%; *P* = 0.006) compared with patients in the true occlusion group. The mortality rate was also higher in PO patients compared with the true occlusion patients (31.6 vs. 14.9%; *P* = 0.006). Likelihood of hemorrhagic complications was similar between the two groups.

### Propensity score matching analysis

Propensity score-adjusted characteristics and outcomes are shown in Table 1. Following 1:1 matching, 36 pairs from each of the PO and true occlusion groups were matched based on similarities in demographic and clinical characteristics. After matching, all covariates showed no significant differences between the two groups. There was an increased likelihood of recanalization failure (OR, 3.18; 95% CI, 1.06–9.59; *P* = 0.040) and poor prognosis (OR, 2.80; 95% CI, 1.07–7.30; *P* = 0.035) in PO patients compared with true occlusion patients (Tables 2, 3). The incidence rates of hemorrhagic complications and mortality were statistically indistinguishable between the two groups after matching.

**Table 2:** Associations between the cervical ICA PO (vs. TO) and reperfusion in the univariate-unadjusted analysis, multivariate-adjusted analysis, and propensity score analyses.

**Variable**	**Number**	**Reperfusion failure, *n* (%)**	**OR (95% CI)**	***P*-value**
**No. of events**				
Cervical ICA PO	79	24 (30.4)		
Cervical ICA TO	67	10 (14.9)		
Univariate-unadjusted analysis	146	112 (76.7)	2.49 (1.09–5.68)	0.031
Multivariate-adjusted analysis^a^	146	112 (76.7)	2.55 (1.07–6.09)	0.036
With matching by propensity score	72	52 (72.2)	3.18 (1.06–9.59)	0.040

ICA, internal carotid artery; PO, pseudo-occlusion; TO, true occlusion; OR, odds ratio; CI, confidence interval. ^*a*^Odds ratio was calculated from the multivariable-adjusted logistic regression models in sensitivity analysis.

**Table 3:** Associations between the cervical ICA PO (vs. TO) and clinical outcome in the univariate-unadjusted analysis, multivariate-adjusted analysis, and propensity score analyses.

**Variable**	**Number**	**Poor outcome, *n* (%)**	**OR (95% CI)**	***P*-value**
**No. of events**				
Cervical ICA PO	79	51 (64.6)		
Cervical ICA TO	67	28 (41.8)		
Univariate-unadjusted analysis	146	79 (54.1)	2.54 (1.30–4.96)	0.006
Multivariate-adjusted analysis^a^	146	79 (54.1)	2.48 (1.08–5.67)	0.032
With matching by propensity score	72	39 (54.2)	2.80 (1.07~7.30)	0.035

ICA, internal carotid artery; PO, pseudo-occlusion; TO, true occlusion; OR, odds ratio; CI, confidence interval. ^*a*^Odds ratio was calculated from the multivariable-adjusted logistic regression models in sensitivity analysis.

### Sensitivity analysis

**Figure 3:**
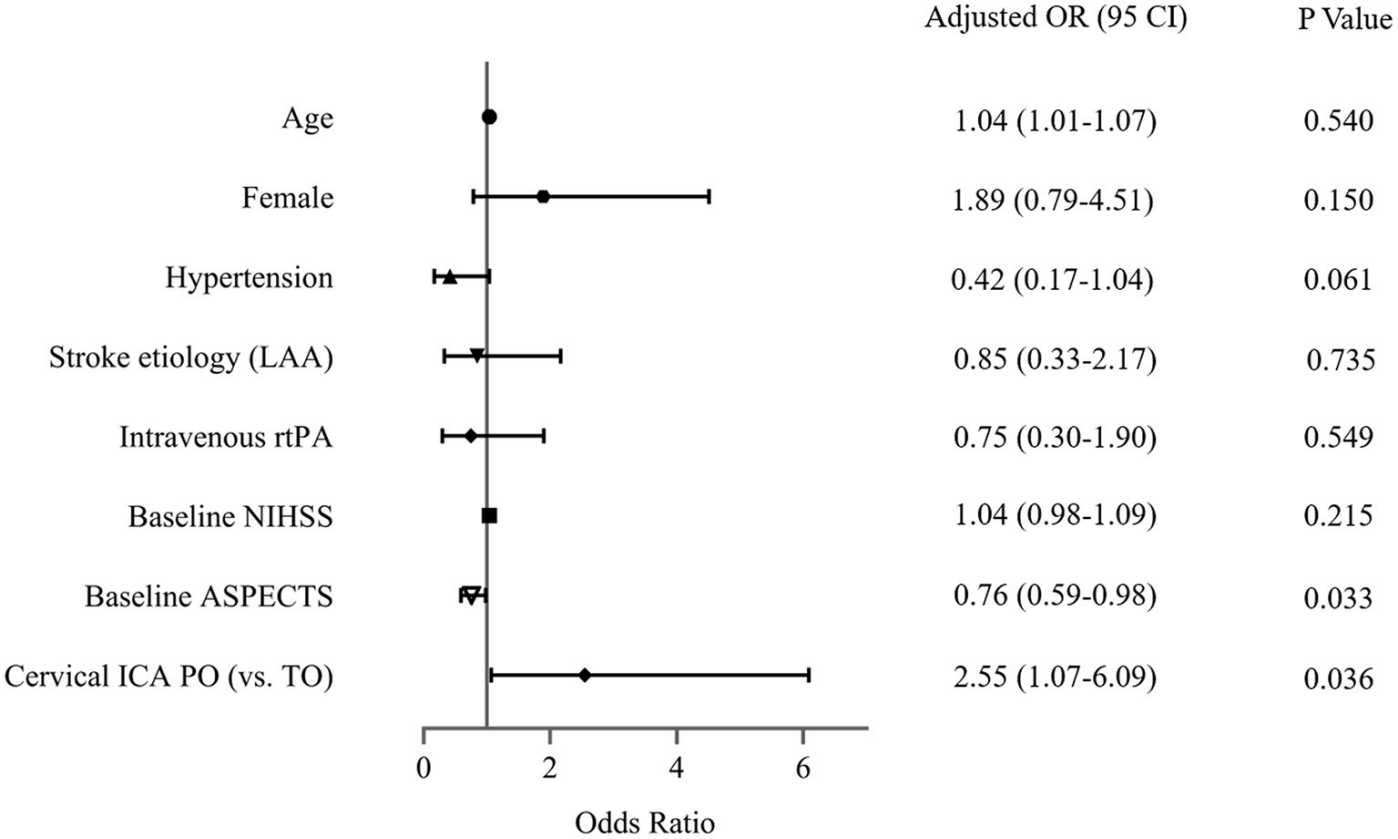
Assessment of association between cervical internal carotid artery (ICA) pseudo-occlusion (PO) and reperfusion in the sensitivity analysis. LAA, large arterial atherosclerosis; rtPA, recombinant tissue plasminogen activator; NIHSS, National Institutes of Health Stroke Scale; ASPECTS, Alberta Stroke Program Early CT Score; TO, true occlusion.

**Figure 4:**
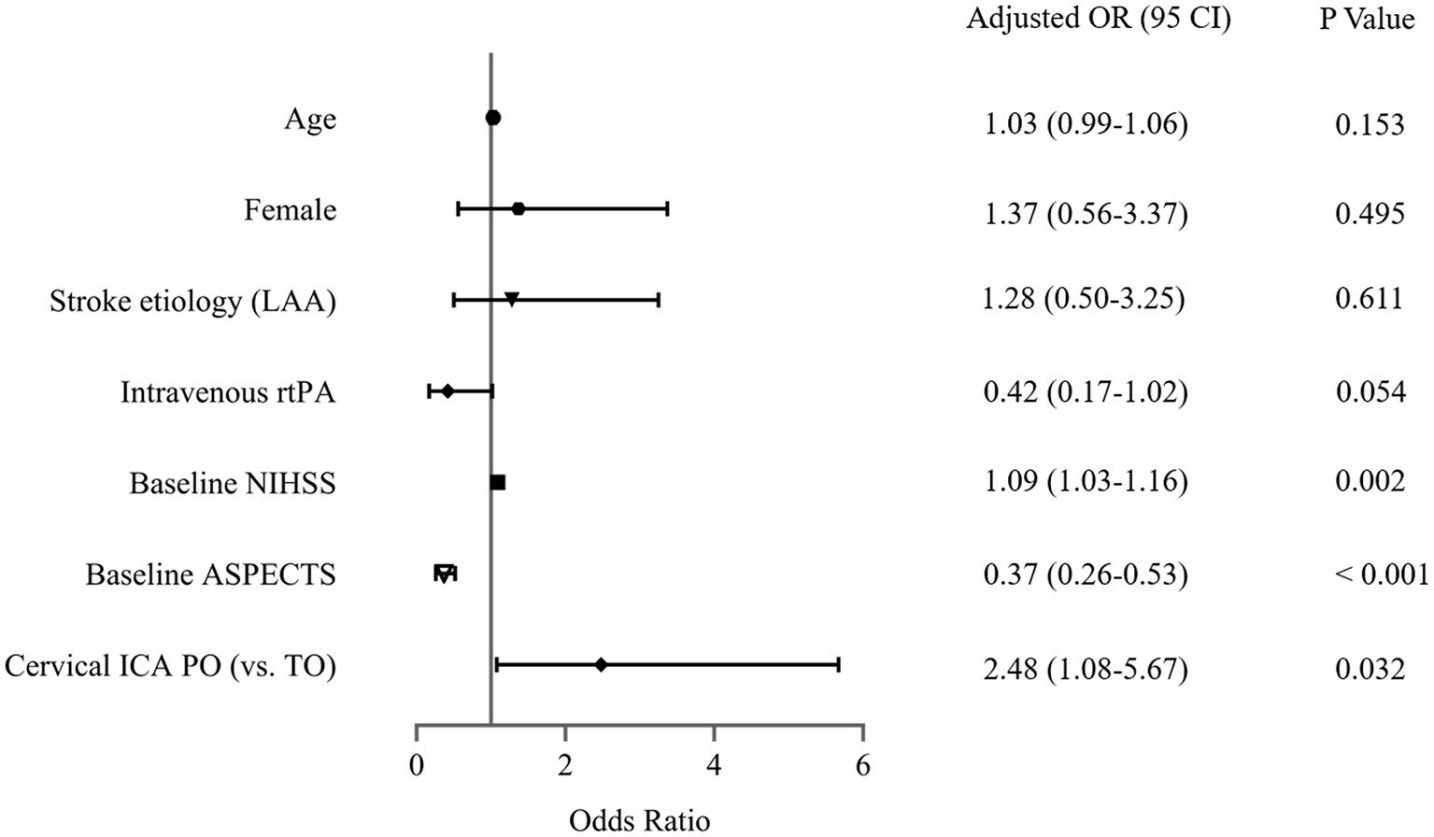
Assessment of association between cervical internal carotid artery (ICA) pseudo-occlusion (PO) and clinical outcome in the sensitivity analysis. LAA, large arterial atherosclerosis; rtPA, recombinant tissue plasminogen activator; NIHSS, National Institutes of Health Stroke Scale; ASPECTS, Alberta Stroke Program Early CT Score; TO, true occlusion.

The sensitivity analysis examines the robustness of the results in the primary analysis. In the multivariable model (Figure 3), the association between cICA PO (vs. true occlusion) and reperfusion (OR, 2.55; 95% CI, 1.07–6.09; *P* = 0.036) was consistent with the main findings after adjustment of confounders (including age, gender, hypertension, stroke etiology, Intravenous rtPA, NIHSS, and baseline ASPECTS). In addition, the OR of the main variable (PO vs. true occlusion) with respect to poor outcome did not change substantially in the multivariable-adjusted model (OR, 2.48; 95% CI, 1.08–5.67; *P* = 0.032) (Figure 4).

## Discussion

In this cohort of patients with cICA occlusion on CTA, we identified a PO incidence of 54.1% based on angiographic exploration. We further found that patients with PO had an increased risk of reperfusion failure and poor outcome compared with true occlusion patients after PSM, and this finding remained robust in the sensitivity analysis. Our study suggests that physicians need to be well aware of the PO phenomenon with greater treatment challenges and determine optimal procedure planning to improve patient prognosis.

Different from the true occlusion, the underlying mechanism of PO may be attributed to the significant hemodynamic changes and sluggish arterial flow caused by acute distal intracranial occlusion (3). Although several imaging techniques (4, 13–16), such as multiphasic CTA and four-dimensional CTA, have been proposed to overcome the low diagnostic accuracy of single arterial-phase CTA, DSA remains the gold standard for differentiating PO from true occlusion. Notably, Grossberg et al. (10) observed that about one-third of patients continued to have a PO on the initial DSA angiogram. They suggested that intraoperative exploration using the microcatheter should be the best way to diagnose the occlusion location and further confirm the presence of PO. Previous studies reported the incidence of PO to be 11–56.3% as confirmed by DSA (5, 6, 10, 11). The prevalence of PO in our series was in line with the findings in the research above. In addition, as suggested by Kappelhof et al. (5) we classified the high-grade atherosclerotic stenoses as true atherosclerotic occlusions in this study because of the similar approach during endovascular treatment.

It has been accepted that successful revascularization is an important determinant of good outcome in patients with acute ischemic stroke (17). Two recent studies showed that patients with PO were more likely to have lower recanalization rates (65.0–71.4%) than those without PO (90.3–100.0%) after thrombectomy (10, 11). Likewise, Chen et al. (9) reported a lower recanalization rate of 36.8% in PO patients compared with non-PO patients (62.7%) after the treatment of intravenous thrombolysis. In this study, we found that patients with PO had an increased likelihood of recanalization failure compared with true occlusion patients. This further supports the fact that PO is more challenging to treat during reperfusion therapy. In the PO phenomenon, local stagnation of blood flow may lead to further enlargement of the original thrombus, making the recanalization process more difficult (9). On the other hand, Kappelhof et al. (5) showed that carotid T occlusion was common in PO patients, as similar to our study. The heavy clot burdens in most PO cases could explain the relatively lower recanalization rate (18, 19). In contrast, the stroke etiology of large artery atherosclerosis was observed more frequently in the cICA true occlusion group. Several studies have shown that combining acute angioplasty with endovascular thrombectomy may increase the successful recanalization rate in the large artery atherosclerosis stroke subtype (20–22). Therefore, accurate evaluation of occlusion type and clot burden before endovascular procedures could help determine the optimal therapeutic planning, which may promote better outcomes (23).

In this study, the rates of unfavorable outcome and mortality were higher in PO patients than those with true occlusion. We also observed that the PO patients had a more severe stroke on admission, which may be related to large clots occluding the terminal ICA segment and leading to insufficient collateral circulation (19). Furthermore, Guglielmi et al. (24) showed that patients with acute stroke caused by cervical carotid atherosclerosis may have more extensive collaterals and better outcomes than patients with cardioembolic stroke. In addition, a relatively higher sICH rate was seen in the PO patients, indicating that more aggressive postoperative management and blood pressure control should be needed in these patients. The poor recanalization rate of the PO patients mentioned above also predetermined a worse clinical outcome. Thus, rapid identification and optimization of treatment strategies on the basis of knowledge of PO should be achieved to ultimately improve reperfusion and clinical outcome.

Several potential limitations should be mentioned in this study. First, it has the inherent limitations of its retrospective design. Second, this study may have a potential risk of ascertainment bias, given the different stroke imaging protocols during the study period. Third, several variables were unavailable in some patients and were not analyzed in this investigation, such as baseline perfusion parameters. In addition, the lack of independent core laboratory adjudication for imaging parameters may affect the reliability of the results. Finally, the modest sample size in a single center may limit the interpretation and generalization of the results. A large and multicenter study is needed to further confirm the results of this study.

## Conclusions

This study suggests that cICA PO is a common phenomenon in patients with large vessel occlusion stroke and may be associated with an increased risk of reperfusion failure and poor outcome after thrombectomy compared with true occlusion. Rapid identification and procedure planning optimization for PO patients should be performed to improve reperfusion and clinical outcome.

## Data availability statement

The raw data supporting the conclusions of this article will be made available by the authors, without undue reservation.

## Ethics statement

The studies involving human participants were reviewed and approved by the ethical standards of the Institutional Research Committee of Jiangsu Province Hospital, The First Affiliated Hospital of Nanjing Medical University. Written informed consent for participation was not required for this study in accordance with the national legislation and the institutional requirements.

## Author contributions

HN and TY analyzed the data and drafted the manuscript. SL designed the study and helped revise this manuscript. LZ conceived the study and made final approval of this manuscript. ZJ and HS helped perform the analysis with constructive discussions. All authors contributed to this article and approved the submitted version.
